# Rapid Ascending Sensorimotor Paralysis, Hearing Loss, and Fatal Arrhythmia in a Multimorbid Patient due to an Accidental Overdose of Fluoxetine

**DOI:** 10.1155/2017/5415243

**Published:** 2017-10-16

**Authors:** Matthew Herrmann, Prissilla Xu, Antonio Liu

**Affiliations:** White Memorial Medical Center Department of Internal Medicine, Los Angeles, CA, USA

## Abstract

**Background:**

Common side effects of selective serotonin reuptake inhibitors (SSRIs) include tachycardia, drowsiness, tremor, nausea, and vomiting. Although SSRIs have less toxic side effects compared to more traditional antidepressants, serious and life threatening cases of SSRI overdose have been reported. We describe a 24-year-old multimorbid female who presented to the emergency department with rapid onset ascending sensorimotor paralysis, complicated by respiratory and cardiac arrest, found to have fatal levels of fluoxetine by toxicological analysis, not taken in a suicidal act.

**Results:**

Autopsy was performed at the Los Angeles County Medical Examiner's Office of a female with no evidence of traumatic injury. Toxicological analysis revealed lethal levels of fluoxetine, toxic levels of diphenhydramine, and multiple other coingested substances at nontoxic levels. Neuropathological examination of the brain and spinal cord revealed no evidence of Guillain-Barre paralysis.

**Conclusions:**

Lethal levels of fluoxetine and multiple potential drug-to-drug interactions in our patient likely contributed to her unique signs and symptoms. This is the first case reporting neurologic signs and symptoms consisting of rapid onset ascending sensorimotor paralysis, hearing loss, respiratory failure, cardiac arrest, and death in a patient with lethal levels of fluoxetine.

## 1. Introduction

Selective serotonin reuptake inhibitors (SSRIs) are commonly used to treat depression and anxiety disorders and are some of the most widely prescribed antidepressants today [1]. Multiple case studies have reported that SSRIs are associated with a less toxic side effect profile and fewer reported deaths have been attributed to overdose compared to more traditional antidepressants, such as tricyclic antidepressants [2, 3]; however, serious and life threatening sequelae have been reported. Additionally, among SSRIs, fluoxetine has been reported to be the least toxic by hazard index measures [3]. The largest published case series on fluoxetine overdoses found that the most common effects were signs of serotonin syndrome such as tachycardia, drowsiness, tremor, nausea, and vomiting [4]. Other significant sequelae include seizures, cardiac toxicity, and death [2, 5]. We report abnormal symptomatology of a young woman due to an accidental fatal fluoxetine overdose, consisting of rapid onset, ascending sensorimotor paralysis, bilateral hearing loss, respiratory failure, cardiac arrest, and eventual death.

## 2. Case Report

A 24-year-old Hispanic female with diabetes mellitus type 1, anemia, hypertension, chronic pancreatitis with partial pancreatectomy, cholecystectomy, and splenectomy presented to the emergency department (ED) with abdominal pain not relieved by oral pain medications. She recalled her home medications to be mirtazapine 30 mg orally nightly, fluoxetine unknown dose orally three times a day, carbamazepine 200 mg orally twice daily, insulin glargine 15–20 units subcutaneously nightly, morphine sulfate 30 mg orally twice daily, tramadol 50 mg orally four times a day, hydromorphone 4 mg orally six times a day as needed for pain, and hydrocodone/acetaminophen 325/10 mg orally six times a day as needed for pain, and she had been taking them to relieve her pain. She was discharged from the ED but returned on the same day with worsening abdominal pain, loss of sensation in lower extremities, lips, and hands, and ascending paralysis. The patient was noted to have a leukocytosis with bandemia, fever, and tachycardia suspicious for sepsis. Computed tomography (CT) of the abdomen was significant for small bowel edema and ascites. The patient continued to deteriorate with worsening ascending paralysis, bilateral hearing loss, hypotension, and respiratory failure with subsequent endotracheal intubation. She underwent two rounds of cardiopulmonary resuscitation (CPR) for a total of approximately 109 minutes but ultimately expired.

An autopsy was performed and documented a well-developed woman with no evidence of traumatic injuries. Postmortem serum analysis revealed fatal levels of heart blood fluoxetine concentration of 2.3 mcg/mL.

The cause of death was thought to be multiple medication intoxication with fatal levels of fluoxetine. A neuropathologist was consulted who agreed with the diagnosis and thought that her symptoms were primarily due to overmedication with fluoxetine. She had no known history of suicidal ideation or attempt but did have chronic pain. Thus, the favored mode of death was accident. Contributory factors to death included acute on chronic, culture negative pancreatitis with abscess formation, and likely sepsis.

## 3. Experimental

### 3.1. Specimens

All specimens were analyzed and collected at autopsy at Los Angeles County Medical Examiner's Office. Submitted specimens included heart ventricles and septum, left lung, right lung, liver, right kidney, small bowel and colon, and head of pancreas. The neuropathological specimens consisted of formalin fixed brain, spinal cord with attached dura mater, and complete cranial dura mater.

## 4. Results

Positive findings on gross and microscopic pathology revealed evidence of chronic pancreatitis with superimposed acute pancreatitis with abscess, culture negative for three days after death, and surgical absent pancreatic tail. The spleen and gall bladder were also surgically absent. The cut surface of the pancreas was pale, fibrotic, and gritty, with loss of normal lobular appearance. Rare small punctate hemorrhages were present, with a single ~0.5 cm possible abscess cavity present centrally in fibrotic area with no communication to the surface. Other microscopic findings revealed acute pneumonia of the left lung, mild emphysematous changes of bilateral lungs, few hypertrophic myocytes of the heart septum and left ventricle, and mild nonspecific chronic hepatitis. There was no evidence of septic emboli.

Gross impression of neuropathological specimens revealed brain swelling, cavum septum pellucidum, external rotation of left hippocampus, beaking of the inferior frontal lobe, cerebral vermis atrophy, and no gross atrophy of the nerves and spinal cord, with no spinal cord lesions noted on cross section. The meninges were clear and there was no gross evidence of meningitis. Neuropathological examination of the brain and spinal cord revealed no evidence of Guillain-Barre paralysis and findings consistent with hypoxic-ischemic encephalopathy. The findings of cerebellar atrophy and hippocampal neuronal dropout suggested a chronic seizure disorder, which was not noted on history.

### 4.1. Toxicological Analysis

Toxicology tests revealed a lethal level of fluoxetine, 1.8 mcg/mL, in the femoral blood, and 2.3 mcg/mL in the heart blood, as well as 0.81 mcg/mL of norfluoxetine in the femoral blood and 1.1 mcg/mL of norfluoxetine in the heart blood. Diphenhydramine was also found at toxic levels, 0.78 mcg/mL, in the femoral blood. Other medications present at nontoxic levels included metoclopramide, mirtazapine, nortramadol, tramadol, midazolam, lidocaine, carbamazepine, hydrocodone, codeine, morphine, and hydromorphone in her heart and femoral blood. Levels of ethanol, barbiturates, cocaine and metabolites, fentanyl, methamphetamine and methlenedioxymethamphetamine, free phencyclidine, free oxycodone, and free oxymorphine were not detected

## 5. Discussion

Fluoxetine, one of the SSRI antidepressants, was introduced into clinical practice over 25 years ago and has remained one of the most popular and safe [6–8] antidepressants in the United States [9]. While approximately half of fluoxetine intoxications remain asymptomatic [4, 9], symptoms of fluoxetine overdose are “minimally toxic” and include tachycardia, drowsiness, tremor, nausea, and vomiting [10]. However, more serious sequelae have also been reported, such as seizures [10–16], cardiac conduction abnormalities [16], CNS depression, respiratory arrest, and even death [5]. Our patient whose toxicological analysis revealed lethal levels of fluoxetine presented with tachycardia and, uniquely, loss of sensation in her lower extremities, lips, and hands and developed rapidly ascending paralysis with respiratory arrest, cardiac arrest, and eventual death.

The patient reported taking fluoxetine, unknown dose, three times a day despite the recommended dose of between 20 and 80 mg per day [17]. It was unclear for how long she had been taking this dose. There was no indication from her history or from the pathologist's report that this was a suicidal attempt. Mode of death determined to be accidental from multiple medication intoxication. The toxicologist report revealed multiple drugs present in her femoral and heart blood, with diphenhydramine, fluoxetine, and norfluoxetine at toxic levels. Our review of the literature revealed that our patient presented with neurologic findings that are first to be described in association with fluoxetine ingestion.

The pathomechanism by which toxic levels of fluoxetine could have caused these unique symptoms is described in, in vitro, animal and human studies. Multiple experimental studies have showed that at hypertherapeutic and overdose concentrations of fluoxetine, fluoxetine demonstrates evidence of cytotoxicity, antiproliferative effects, and mitochondrial dysfunction. Specifically, these studies noted inhibition of mitochondrial function and depletion of cellular ATP levels with significantly increased lactate production, activation of apoptosis, and evidence of redox stress and DNA damage [18–22].

The drug-drug interactions which potentially contributed to our patient's toxic levels of fluoxetine and unique symptomatology include fluoxetine with metoclopramide, carbamazepine, tramadol, mirtazapine, codeine, hydrocodone, and morphine. Fluoxetine, diphenhydramine, and metoclopramide are all inhibitors CYP2D6, one of the cytochrome P450 enzymes necessary for detoxification of foreign chemicals and metabolism of drugs. Inhibitor of this enzyme likely contributed to the toxic levels noted in her blood. Also, it is well documented that the concurrent use of fluoxetine with codeine, hydrocodone, hydromorphone, mirtazapine, morphine, and tramadol, and the use of concurrent carbamazepine with codeine, hydromorphone, and mirtazapine may result in increased risk of serotonin syndrome [23–25]. Overall, our patient's unique symptomatology could be explained by the damaging effects of toxic levels of fluoxetine as well as the multiple drug-drug interactions as outline in Table 1.

Most fatalities due to fluoxetine exposure are reported either with extremely large doses (greater than 150 times the daily dose) or with the presence of coingestants such as ethanol or benzodiazepines [4, 6]. In 2014, the NDPS reported that the range of fluoxetine blood concentrations in listed fatalities ranged from 0.68 mcg/ml to 2.2 mcg/L, which is significantly greater than the typical steady state therapeutic concentration of 0.25 mcg/mL. To put this into perspective, toxicological analysis on our patient revealed fatal levels of heart blood fluoxetine and norfluoxetine concentrations at 2.3 mcg/mL and 1.1 mcg/mL, respectively, well above the therapeutic limit. It is important to note that, in one study of antidepressant overdose, no correlation between fluoxetine level and mental status was found, while another study of fluvoxamine overdose found no correspondence between drug levels and symptom severity [17, 18], suggesting that each patient presenting with an SSRI overdose, despite quantity consumed, can have unique symptomatology.

Although most patients recover from fluoxetine overdose, high dose ingestions can lead to cardiovascular and neurologic complications, including death. In this case, the patient had lethal levels of fluoxetine noted in her blood, as well as levels of multiple other medications with known drug-drug interactions to fluoxetine, possibly contributing to her unique symptomatology. However, this patient's presenting neurologic signs and symptoms are unique and have yet to be described in current reports of fatal levels of fluoxetine ingestions or coingestions involving fluoxetine.

## Figures and Tables

**Table 1:** Drugs found in patient's blood by toxicological analysis, site of metabolism, reference range, and documented drug-drug interactions.

Medication	Blood site	Measured concentration (mcg/mL)	Metabolism	Reference range	Drug-drug interactions	Comment
*Diphenhydramine*	Femoral/heart	*0.78/0.63* *(toxic level)*	50% liver metabolism to diphenylmethane, which suggests a large first-pass effect	Antihistamine effects at levels >0.025 mcg/mL Drowsiness at levels 0.03–0.04 mcg/mL *Mental impairment at levels >0.06 mcg/mL* *Toxic: >0.1 mcg/mL* Therapeutic: not established	Metabolism/transport effects inhibit CYP2D6 (moderate) and CYP1A2, 2C9, and 2C19 (minor)	*May increase fluoxetine level via inhibition of CYP2D6*

*Fluoxetine*	Femoral/heart	1.8/2.3 *(toxic level)*	CYP450 (extensive P450 CYP2D6 inhibitor, demethylation) Active metabolite: norfluoxetine	Therapeutic: fluoxetine: 0.1–0.8 mcg/mL Toxic: fluoxetine plus norfluoxetine: >2 mcg/mL	Metabolism/transport effects substrate of CYP1A2 (minor), 2B6 (minor), 2C9 (major), 2C19 (minor), 2D6 (major), 2E1 (minor), and 3A4 (minor) It inhibits CYP1A2 (moderate), 2B6 (weak), 2C9 (weak), 2C19 (moderate), 2D6 (strong), and 3A4 (weak)	Avoid concomitant use with MAO inhibitors *Increased effect/toxicity of serotonin reuptake inhibitor/antagonist, carbamazepine, CNS depressants, CYP1A2 substrates, CYP2C19 substrates, CYP2D6 substrates*, serotonin modulators

*Norfluoxetine*	Femoral/heart	0.81/1.1 *(toxic level)*	Active metabolite of fluoxetine	Therapeutic: norfluoxetine (active metabolite): 0.1–0.6 mcg/mL Toxic: fluoxetine plus norfluoxetine: >2.0 mcg/mL		*Patient had combined fluoxetine + norfluoxetine 3.3 mcg/ml in heart blood, over the toxic limit 2.0 mcg/ml*

Metoclopramide	Femoral/heart	0.10/0.10	Hepatic: minimal, via simple conjugation	N/A	Metabolism/Transport effects substrate of (minor) CYP1A2, 2D6; it inhibits CYP2D6 (weak)	*May increase fluoxetine level via inhibition of CYP2D6*

Mirtazapine	Femoral/heart	0.19/0.11	CYP450 extensively hepatic via CYP2D6, CYP1A2, CYP2C9, and CYP3A4 Metabolites: 8-hydroxyl metabolite, N-desmethyl, and N-oxide metabolites	N/A	Metabolism/transport effects substrate of CYP1A2 (major), 2C9 (minor), 2D6 (major), and 3A4 (major) It inhibits CYP1A2 (weak), 3A4 (weak)	*May increase levels/effects of CNS depressants*Serotonin modulators The level/effects of mirtazapine may be increased by CYP1A2 inhibitors (strong), 2D6 inhibitors (moderate), CYP3A4 inhibitors (moderate)

Tramadol	Femoral/heart	0.2/0.17	Extensively hepatic via demethylation, glucuronidation, and sulfation Pharmacologically active metabolite formed by CYP2D6 (M1; O-demethyl tramadol)	0.1–0.3 mcg/mL However, serum level monitoring is not required	Metabolism/transport effects substrate of CYP2D6 (major), 3A4 (major)	*May increase levels/effects of CNS depressants; MAOI; SSRIs*: Serotonin modulators The level/effects of tramadol may be increased by CYP2D6 inducers (moderate), 3A4 inhibitors (strong) The levels/effects of tramadol may be decreased by CYP2D6 inhibitors (moderate), 3A4 inducers (strong)

Nortramadol	Femoral/heart	0.82/0.84	Active metabolite of tramadol	0.1–0.3 mcg/mL However, serum level monitoring is not required		

Lidocaine	Heart	<0.5	CYP450 90% hepatic metabolism via CYP1A2 Active metabolites: Monoethylglycinexylidide (MEGX) Glycinexylidide (GX)	Therapeutic: 1.5–5 mcg/mL Potentially toxic levels: >6 mcg/mL Toxic: >9 mcg/mL	Metabolism/transport effects substrate of CYP1A2 (minor), 2A6 (minor), 2B6 (minor), 2C9 (minor), 2D6 (major), and 3A4 (major); it inhibits CYP 1A2 (strong), 2D6 (moderate), and 3A4 (moderate)	Lidocaine may decrease the levels/effects of tramadol *May increase fluoxetine level via inhibition of CYP2D6*

Midazolam	Heart	0.105	Metabolized in the liver and gut via biotransformation mediated by CYP3A4	N/A	Metabolism/transport effects substrate of CYP2B6 (minor), 3A4 (major) and it inhibits CYP 2C8 (weak), 2C9 (weak), and 3A4 (weak)	*The levels/effects of midazolam may be increased by SSRIs* The levels/effects of midazolam may be decreased by carbamazepine and CYP3A4 inducers (strong)

Carbamazepine	Heart	5.0	CYP450 Primarily via CYP3A4 Active metabolites: Carbamazepine-10,11-epoxide (partly responsible for intoxication)	Therapeutic levels: 4–12 mcg/mL Toxic level: >15 mcg/mL	Metabolism/transport effects substrate of CYP2C8 (minor), 3A4 (major), It induces CYP 1A2 (strong), 2B6 (strong), 2C8 (strong), 2C9 (strong), 2C19 (strong), and 3A4 (strong)	*May increase the levels/effects of CNS depressants* *The levels/effects of carbamazepine may be increased by CYP3A4 inhibitors and SSRIs* (i.e., fluoxetine)

Hydrocodone	Heart	Free hydrocodone level 0.06	Liver metabolism by N-demethylation (catalyzed by CYP3A4, 2B6, and 2C19), 0-demethylation (catalyzed by CYP2B6 and 2C19), and 6-keto reduction to the corresponding 6-alpha and 6-beta hydroxyl metabolites Active metabolites: Norhydrocodone (major) Hydromorphone (minor)	N/A	Metabolism/transport effects substrate of CYP3A4 (major)	*May increase the levels/effects of CNS depressants and SSRIs* (i.e., fluoxetine)

Codeine	Heart	Presumptive+	CYP450 Primarily via CYP2D6, CYP3A4, and UDP-glucuronosyl-transferase 2B7 and 2B4	Therapeutic: not established Toxic: >1.1 mcg/mL	Metabolism/transport effects substrate of CYP2D6 (major) and CYP3A4 (minor) It inhibits CYP2D6 (weak)	*May increase the levels/effects of CNS depressants and SSRIs (i.e., fluoxetine)* *May increase fluoxetine level via CYP2D6 inhibition*

Morphine	Heart	Presumptive+	Liver metabolism by N-demethylation, N-dealkylation, 0-dealkylation, conjugation, and hydrolysis Active metabolite: Morphine-3-glucuronide (M3G) Morphine-6-glucuronide (M6G)	Therapeutic: surgical anesthesia: 65–80 ng/mL Toxic: 200–5000 ng/mL	Metabolism/ transport effects substrate of CYP2D6 (minor)	*May increase the levels/effects of CNS depressants and SSRIs (i.e., fluoxetine)*

Hydromorphone	Heart	Presumptive+	Extensive liver first-pass metabolism primarily via glucuronidation Inactive metabolite: Hydromorphone-3-glucuronide	N/A	Not a significant substrate of inhibitor of CYP450	*May increase the levels/effects of CNS depressants and SSRIs*
